# Mimotope vaccination for epitope-specific induction of anti-VEGF antibodies

**DOI:** 10.1186/1472-6750-13-77

**Published:** 2013-09-27

**Authors:** Weina Li, Yonggang Ran, Meng Li, Kuo Zhang, Xin Qin, Xiaochang Xue, Cun Zhang, Qiang Hao, Wei Zhang, Yingqi Zhang

**Affiliations:** 1 The State Key Laboratory of Cancer Biology, Department of Biopharmaceutics, School of Pharmacy, Fourth Military Medical University, Xi’an, China; 2 Training Department, Bethune Military Medical NCO of PLA, Shijiazhuang, Hebei, China

**Keywords:** Avastin, VEGF, Cancer immunotherapy, Mimotope, Phage display library

## Abstract

**Background:**

Tumor angiogenesis is critical for tumor growth, infiltration and metastasis. Vascular endothelial growth factor (VEGF) is a potent angiogenic factor and targeting it is important in reducing angiogenesis. Bevacizumab (Avastin), a monoclonal antibody that reacts directly against VEGF, has been demonstrated to be an effective treatment for various cancers such as rectal cancer, colon carcinoma, and non-small cell lung cancer, etc.

**Results:**

In the current study, we used the phage display technique to generate mimotopes that complemented the screening Avastin antibody (Ab). The candidate mimotopes of VEGF were isolated from a 12-mer peptide library. The phage displaying peptide DHTLYTPYHTHP (designated as 12P) exhibited high affinity to Avastin. The chemically synthesized 12P was conjugated to keyhole limpet hemocyanin (KLH) by glutaraldehyde (GA) to form vaccine KLH-12 peptide (KLH-12P). This epitope vaccine significantly induced humoral immunity in mice. The blood serum from KLH-12P-immunized mice associated with VEGF and blocked its binding to VEGFR, thus inhibiting vascular endothelial cell proliferation and migration.

**Conclusions:**

Our data indicate that the isolated mimotope 12P reported here could potentially elicit specific antibodies against VEGF and result in the induction of anti-angiogenesis responses.

## Background

Searching for new therapies against cancer is of great importance to human health. Recent therapeutic interventions target various aspects of cancer growth and distant metastasis. The advancement in knowledge of the importance of angiogenesis to tumor progression and metastasis has driven the development of anti-angiogenic therapies for cancer treatment. The concept of anti-angiogenic therapy began more than 30 years ago, when Judah Folkman demonstrated that factors released by tumors mediate angiogenesis [1,2]. Angiogenesis is a necessary step for tumor growth beyond 1–2 mm, as well as for the development of metastasis [3,4].

VEGF, secreted by tumors, was first discovered in 1983 and was later shown to play a crucial role in cancer initiation, progression and angiogenesis stimulation [5]. Since then, therapeutic agents that target VEGF have been developed and tested. It has been shown that an anti-VEGF antibody can reduce the blood vessel density in a given microscopic area in a tumor, termed the microvessel density, and inhibit the growth of some tumors in nude mice [6]. Also, bevacizumab (Avastin), which is a humanized monoclonal antibody that binds VEGF, has been approved for the treatment of cancer. However, these therapeutic antibodies have serious practical limitations [7], including obstacles in production. In addition, antibiotictherapy is expensive and requires repeated administration over a long period of time.

Mimotopes are small peptides that structurally mimic a given antibody-binding site of an epitope and can be recognised by the immune system [8,9]. Active immunization using mimotopes induces antibodies to recognize the mimicked epitope. Becuase mimotopes can produce ongoing immune responses [10], avoid repeated administration, provide affordable medicines, and lead to broader patient acceptance and compliance, they may be a promising next step in drug development. Mimotopes can be isolated from phage display peptide libraries [11] and have been shown to drive active immune responses towards the original antigen, thus leading to effective immunity. They are usually used in the development of vaccines against many kinds of diseases [12-15]. Li reported that mimotopes induced the production of protective antibodies, and consequently, became candidates for the development of potential vaccines [16].

In this study, we investigated alternative anti-angiogenic therapies and exploited phage display technology to identify the peptides that can mimic the natural VEGF epitope. The PhD.-12™ Phage display peptide library was immunoscreened and the selected phage plaques were analyzed. The mimotopes were used to immunize mice and the immune responses for the phagotopes were evaluated.

## Results

### Biopanning and screening

**Figure 1:**
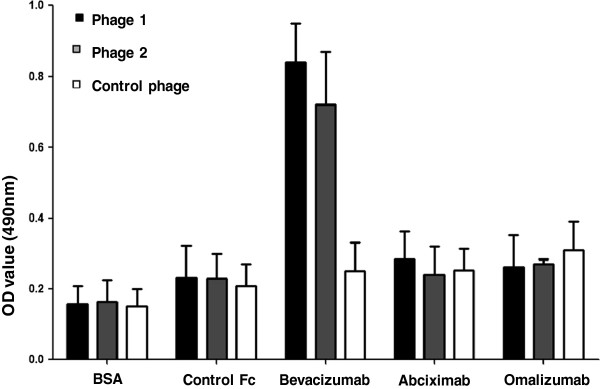
**Binding specificity of selected phages from the Ph.D.-12**^**TM **^**Phage display peptide library.** Wells of 96-well microtitre plates were coated with Avastin mAb, control antibodies or BSA by incubation at 4°C overnight and blocked with 1% BSA in TBS. Affinity-selected phage were added to the wells, allowed to bind at room temperature for 2 h and determined at OD490. Phage 1: phage plaques displaying peptide DHTLYTPYHTHP; Phage 2: phage plaques displaying peptide NHFGKFLDALAG; Control phage: uncombined phage in the first round of panning. All assays were carried out in triplicate and the error bars indicate standard deviation.

A library of random 12 mer peptides was screened with Avastin. As the first indicator of successful biopanning, an increase of phage titers during rounds of panning was observed. The phage titer increased from 1.21 × 10^8^ plaque forming units (pfu)/μL (first round) to 1.02 × 10^9^ pfu/μL (second round) and finally to 7.59 × 10^9^ pfu/μL (third round). A total of 80 phage plaques were randomly chosen for colony screening after three successive rounds of biopanning. Among them, 42 phage plaques with the highest affinity with Avastin, but not with control or the blocking protein (BSA), were found and amplified. All candidates showed comparable binding intensities. The ssDNA from these 42 phage plaques was sequenced and two mimotope candidates (DHTLYTPYHTHP and NHFGKFLDALAG) were specifically recognized by the selecting antibody, Avastin. The DHTLYTPYHTHP insert was the sequence most frequently found, indicating high reactivity of the corresponding peptide with the Avastin paratope. The NHFGKFLDALAG sequence was also deduced from the sequencing result. The positive phage candidates containing these two mimotopes were specifically recognized by Avastin, but not by control antibodies or BSA. Moreover, Avastin did not react with the 12-mer peptide control phage (Figure 1).

### Binding of Avastin to the peptide displayed on phages

**Figure 2:**
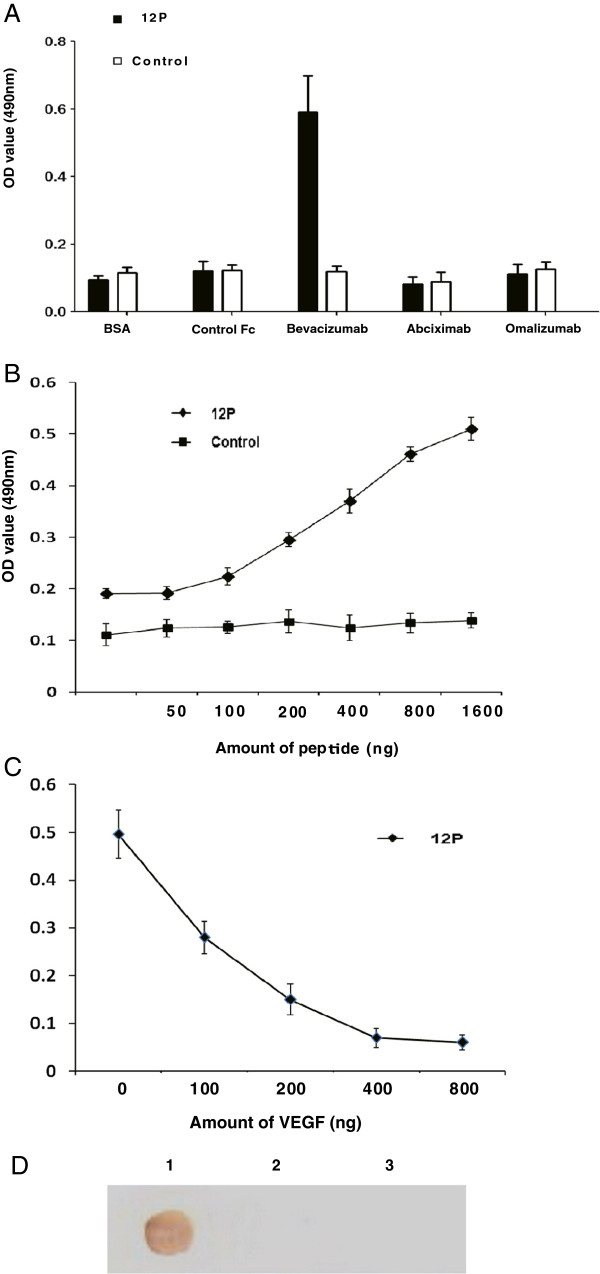
**Synthesized peptide (12P) binding to Avastin. (A)** ELISA of DHTLYTPYHTHP bound to Avastin, the control antibodies and BSA are shown here. Each antibody solution was added to the wells and incubated overnight, after which the wells were blocked with 1% BSA and C-terminally biotin-labeled 12P or control peptide was added. The plates were again washed and HRP-conjugated streptavidin was then used to act with OPD. A representative result of three experiments is shown. **(B)** 12P binding to Avastin is dose-dependent. The 96-well plate was coated with Avastin. The binding ability of 12P (filled diamond) or control (filled square) with Avastin was analyzed. A representative result of three experiments is shown. **(C)** 12P binding to Avastin can be blocked by VEGF. The 96-well plate was coated with Avastin. After blocking with 1% BSA, the plate was incubated with the indicated amount of VEGF and 800 ng 12P per well. A representative result of three experiments iss shown. **(D)** Specific recognition of the synthetic mimotope constructs by Avastin (Dot Blot). Lane 1, 12P-KLH; lane 2, Control peptide-KLH; lane 3, KLH. A representative experiment of three performed is shown.

To determine whether the peptide still has the ability to bind to Avastin upon removal from the phage, DHTLYTPYHTHP (designated as 12P), the most frequently found in positive phage plaques, was chemically synthesized. The mimotope 12P was specifically recognized by Avastin only (Figure 2A). In addition, a dose-dependent increase in the binding of 12P to Avastin was observed viaELISA (Figure 2B). Further experiments revealed that VEGF can compete with 12P to bind to Avastin (Figure 2C).

### Association of mimotope conjugates with Avastin

Dot blot assay was performed to investigate the specific recognition of the synthetic mimotope constructs by Avastin. The antigen 12P-KLH was associated with Avastin (lane 1), but not control peptide-KLH and KLH (lanes 2 and 3, respectively). As shown in Figure 2D, DHTLYTPYHTHP-KLH was the only one of the three KLH conjugates that was specifically recognized by Avastin.

### Binding of serum from vaccinated mice to VEGF protein

**Figure 3:**
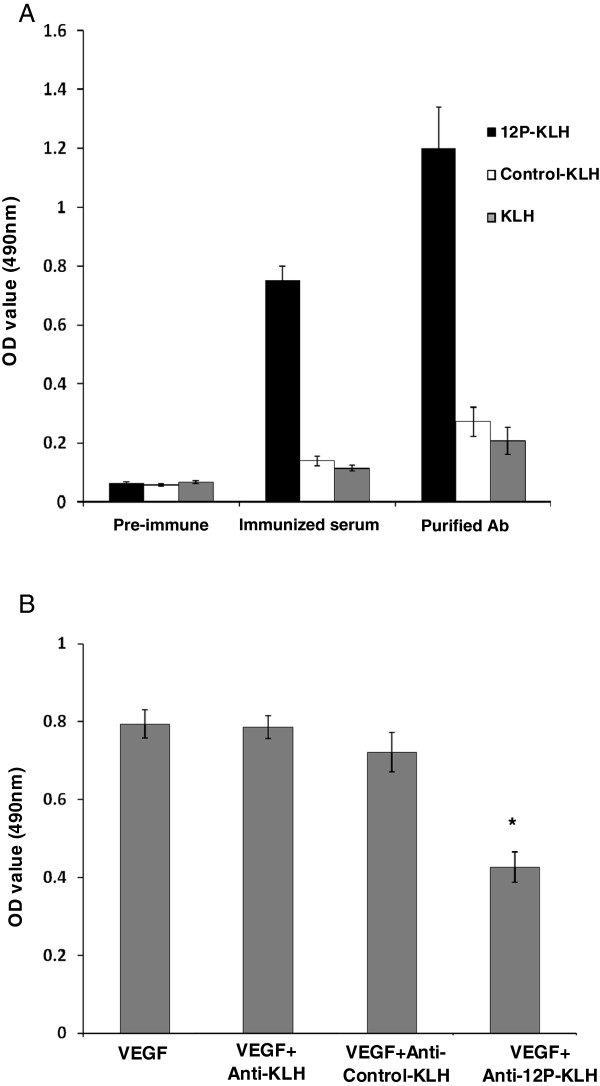
**The immunized mouse serum antibody recognizing VEGF and blocking VEGF binding with VEFGR. (A)** Wells of 96-well microtitre plates were coated with the recombinant VEGF (50 ng/well) and, after blocking, were incubated with the mouse serum: 4% pre-immune serum; 4% serum after immunization or 2 μg/ml purified antibody of immunized mice serum. The antiserum elicited by 12P-KLH could bind to VEGF. This result is representative of three independent experiments. **(B)** Wells of 96-well microtitre plates were coated with the recombinant VEGFR2 (10 ng/well) and then incubated with VEGF with or without purified serum. Bound human VEGF was detected with a rabbit anti-human VEGF and following anti-rabbit IgG conjugated with HRP. All assays were carried out in triplicate and the error bars indicate standard deviation. Statistical analysis was performed using the Student’s t test (*p < 0.05).

In this assay, 12P-KLH, control peptide-KLH and KLH immunogenicity was evaluated in BALB/c mice. All 12P-KLH-immunized mice developed high anti-mimotope titers (>1:100,000), indicating successful immunization in these mice. The pre-immune serum, serum after immunization of 3 groups were collected and purified for further experiment. The antiserum and purified antibody from 12P-KLH-immunized mice recognized the recombinant VEGF protein (Figure 3A). Furthermore, the purified antibody from 12P-KLH immunized mice could block VEGF binding with VEFGR (Figure 3B).

### Antiserum inhibited the proliferation and migration of human umbilical vein endothelial cells

**Figure 4:**
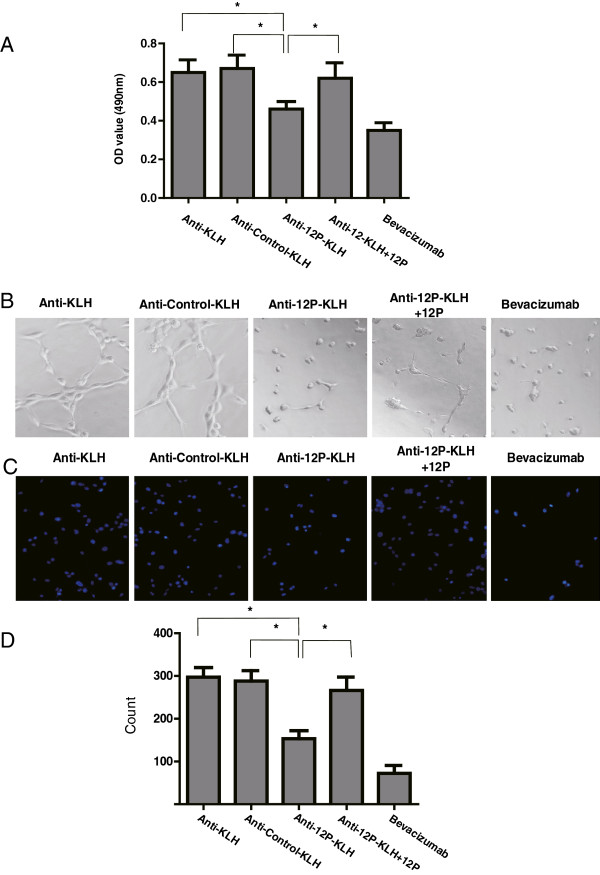
**Antiserum from 12P-KLH-vaccinated mice inhibited proliferation, matrigel tube formation and migration of HUVECs. (A)** MTT assay of vascular endothelial cells treated with the indicated groups: anti-KLH, anti-control-KLH, anti-12P-KLH. 12P was used to block the function of anti-12P-KLH and Avastin was used as the positive control. All assays were carried out in triplicate and the error bars indicate standard deviation. Statistical analysis was performed using the Student’s t-test (*p < 0.05). **(B)** Inhibition of tube formation of HUVECs treated with antibodies was detected on Matrigel. HUVECs (1 × 10^5^/ml) were seeded on top of the matrigel with complete RPMI 1640 medium containing antibodies and cultured for 8 h. The representative photographs were taken using a light microscope (200× view field). **(C)** HUVECs were seeded in the upper chamber of a Millicell insert with RPMI 1640 containing antibodies and incubated with 10% FCS RPMI 1640 medium containing 5 ng/ml VEGF in the lower chamber for 12 h. The cells were fixed and stained with 4,6-diamidino-2-phenylindole (DAPI). The migrated cells were visualized under a microscope. A representative image of DAPI staining is shown. **(D)** Data of C was analyzed by Student’s t-test. *p < 0.05. All of the assays were carried out in triplicate and the error bars indicate standard deviation.

We further assessed the effect of anti-12P-KLH antibodies on the apoptosis of vascular endothelial cells. Methyl thiazolyl tetrazolium (MTT) analysis showed that the purified antibody from 12P-KLH immunized mice inhibited the proliferation of HUVECs. This effect was blocked by the 12P peptide (Figure 4A). Tube formation was also decreased by the purified antibody from 12P-KLH immunized mice. Consistent with the above result, 12P peptide abolished the inhibition of tube formation effect of anti-12P-KLH (Figure 4B). Migration assay results showed that the migrated cells were 297 ± 22.7 (Anti-KLH), 288 ± 24.5 (Anti-Control-KLH), 153 ± 19.3 (Anti-12P-KLH), 266 ± 31.6 (Anti-12P-KLH ± 12P) and 72 ± 18.9 (Avastin), respectively (Figure 4C,D). The mobility of the HUVECs was significantly lower in the anti-12P-KLH and Avastin groups compared to the other three groups.

## Discussion

The phage-displayed technique is a useful tool in selecting small peptides that mimic antigens. Peptide mimotopes can bind to antibodies which are raised against native structures. The strategy of using obtained peptide mimics (mimotopes) as low molecular weight substitutes of the natural antigen for active immunization could potentially elicit specific antibodies and result in the induction of long-lasting humoral immune responses. In the case of mimotope immunization, several studies have shown effective responses in vivo [17]. Importantly, the mimotopes are able to replace the original epitopes for vaccine development [18,19]. Furthermore, active immune responses induced by phage-displayed mimotopes have been verified in many diseases [20-22]. Mimotopes can potentially serve as lead compounds in the development of low molecular weight substitutes of the template protein for use in drug and vaccine design [23]. This may be an effective strategy to overcome the serious practical limitations of the therapeutic monoclonal antibodies, e.g. Avastin.

In this study, a library of random 12 mer peptides was used to identify the VEGF epitope (mimotope) using Avastin as a selector molecule. After three rounds of biopanning, two amino acid sequences were deduced from these phage plaques. We isolated two peptide mimics (DHTLYTPYHTHP and NHFGKFLDALAG) of the epitope that were specifically recognized by the Avastin mAb. We aligned the mimotope sequences and did not observe any homology between the two mimotopes and the known amino acid sequence of VEGF. Many researchers have demonstrated similar results to ours. One reason for the disparity between the two mimotopes and VEGF might be that the epitopes that are recognized by Avastin could be a discontinuous amino acid sequence due to the folding of peptides, thus forming a conformational epitope [19]. MimoPro is a new graph-based mapping method for epitope prediction using affinity-selected peptides derived from phage display experiments and is freely accessible through the MimoPro server [24]. We used MimoPro to analyze the two newly isolated peptide mimics, as well as mimotope M074_D12 [25]. As shown in Additional file 1: Figure S1, the mimotope 12P (DHTLYTPYHTHP) had the highest score among the three, suggesting that 12P shares the most similar structure with VEGF.

To demonstrate the affinity between the insert sequences displayed on positive phage plaques and Avastin, the results of colony screening were confirmed by ELISA. 12P was specifically recognized by the selecting antibody Avastin, but not by the control antibody or BSA. In addition, the binding of 12P to Avastin was observed in a dose-dependent manner. Most importantly, VEGF could compete with 12P in binding to Avastin.

Due to immune tolerance, the use of a natural epitope peptide alone is insufficient for inducing an immune reaction [26]. Therefore, we conjugated the 12P peptide to KLH to form the antigen. The conjugate vaccine proteins were then applied for Dot blot analysis. We found that 12P-KLH was associated with Avastin, but not control-KLH or KLH. This indicated that 12P-KLH was the only conjugate vaccine of the three peptides that could be specially recognized by Avastin.

Titer determination was performed to evaluate the immunogenicity of the conjugations. Mice immunized with KLH and control-KLH only developed antibodies against KLH but not VEGF. All mice immunized with 12P-KLH successfully developed high titers of antibodies against VEGF as well as the KLH, indicating successful immunization in mice.

It was crucial to determine whether the anti-12P-KLH antibodies could bind to the VEGF protein. Therefore, we performed experiments to demonstrate whether the immunized serum was specific for VEGF. Our results demonstrated that the conjugated vaccine containing 12P mimicked the natural VEGF epitope and was capable of inducing an active, specific antibody response to VEGF. Altogether, our findings showed that the phage-displayed epitope was able to mimic immunological properties of the native epitope on VEGF. Furthermore, using 12P-KLH as the antigen, we were able to detect the antibody in the serum from immunized animals and the latter could block VEGF binding to VEGFR2.

Without new blood vessels and a functional vasculature, tumors are limited in their ability to grow and tumor cell proliferation would give way to apoptosis. The purpose of our study was to generate mimotope vaccines that could induce the generation of antibodies against VEGF. We further assessed the effect of purified anti-12P-KLH antibodies on the vascular endothelial cells (HUVECs). Decreased proliferation of HUVECs after treatment with anti-12P-KLH antibodies was observed, compared with equal amounts of anti-control-KLH or anti-KLH antibodies. Tube formation and cell migration were also decreased by anti-12P-KLH antibodies. The anti-12P-KLH antibody-induced effects were abolished by the 12P peptide. Our findings showed that the anti-12P-KLH antibodies could remarkably block the tube formation, proliferation and migration induced by VEGF on HUVECs.

The strategy of generating 12P-KLH vaccine constructs for active immunization has many advantages. For example, the mimotopes are easily synthesized and conjugated, and can actively induce continuously available antibodies against VEGF. Our results suggest that the phagotope 12P provides a convenient and economic approach to preparing antigens, and could be used in anti-angiogenic therapy.

## Conclusions

The commercialized antibody, Avastin, is a promising anti-tumor agent that recognizes VEGF and suppresses angiogenesis. To overcome its limitations, we generated epitope mimics that bind to Avastin and tested whether the peptide mimics could induce the production of similar antibodies upon active immunization of mice with the peptides. We used the Ph.D-12 phage display peptide library to identify peptides that bind to Avastin. Two mimotopes (DHTLYTPYHTHP and NHFGKFLDALAG) were shown to bind to the mAb. The most frequently selected mimotope, DHTLYTPYHTHP (12P), was conjugated to KLH and used to immunize BALB/c mice. Antiserum from 12P-KLH-immunized mice could recognize the recombinant VEGF, whereas sera from mice immunized with the control peptide-KLH or carrier KLH alone failed to show similar reactivity. Furthermore, anti-12P-KLH antibodies induced the inhibition of tube formation, proliferation and migration of HUVECs. Our data indicate that the isolated mimotope 12P reported here could potentially elicit specific antibodies against VEGF and result in the inhibition of human umbilical vein endothelial cells.

## Methods

### Cell lines, animals and monoclonal Abs

The human umbilical vein endothelial cells (HUVECs), purchased from Shanghai Institutes for Biology Sciences of the Chinese Academy of Sciences, were routinely cultured in monolayers at 37°C with 5% CO_2_. Cells were maintained in RPMI 1640 medium (Gibco, USA) supplemented with 10% heat-inactivated fetal bovine serum, 100 μg/ml ampicillin and 100 μg/mL streptomycin. Seven-week-old healthy female BALB/c mice, purchased from the National Rodent Laboratory Animal Resource (Shanghai, China), were cared for under institutional animal care protocols. Bevacizumab (Avastin) was purchased from Roche (Switzerland). Abciximab (ReoPro) and Omalizumab (Xolair) were purchased from Eli Lilly (Switzerland). Isotype-matched human IgG1 Fc control was purchased from R&D (USA). Both rhVEGFA and rhuman VEGFR were purchased from Sino Biological Inc. (Beijing, China). Anti-human VEGF was obtained from ABcam (England).

### Phage library and biopanning

A total of three successive rounds of panning with Avastin were performed with a random phage display 12-mer peptide library (BioLabs, New England) that expressed random 12-mer peptides at the N-terminus of the minor M13 phage coat protein. The procedure for screening the phage display library was performed according to instruction of the manufacturer of the kit. Briefly, 96-well ELISA plates were coated with 20 μg/well of Avastin overnight at 4°C. Wells were blocked for 2 h with 1% bovine serum albumin (BSA) (Sigma, USA) in TBS and incubated with an aliquot of the phage library (2 × 10^11^ phage particles) in binding buffer (TBS, 0.1% Tween-20 or 0.5% Tween-20). Unbound phage particles were removed by extensive washing with TBS/0.5% Tween 20. Bound phages were eluted with 0.2 M Glycine–HCL/0.1% BSA, pH 2.2 and immediately neutralized. Phages were amplified in *Escherichia coli* ER2738, precipitated from the bacterial culture supernatant with polyethylene glycol, and then titered before the next round of biopanning. For titer determination, aliquots of the elution or amplification phases were plated in serial dilutions on Luria broth agar plates containing 20 μg/mL of tetracycline.

### Phage plaques screening assay

After three rounds of biopanning, screenings for the selection of specific phage plaques were completed. Immunoscreenings were performed with Avastin and control. Wells were coated with Avastin (100 ng/well) overnight at 4°C. After blocking, phage plaques (1 × 10^9^/well) were added to the wells and incubated for 2 h at room temperature. Wells were washed 10 times with TBS/0.5% Tween 20 and bound phage was detected by HRP-conjugated anti-M13 monoclonal antibody (Amersham Biosciences, Germany). The reaction was developed with OPD (Sigma, USA). The absorption value was measured at 490 nm with a Bio-Rad ELISA reader. Positive phage plaques were amplified and identified by DNA sequencing.

### Specificity enzyme-linked immunosorbent assay

ELISA plates were incubated with Avastin or control antibodies (100 ng/well) overnight at 4°C. Plates were then washed with PBS containing 0.1% Tween 20 and blocked with PBS containing 0.1% Tween 20 and 1% BSA prior to the addition of phage particles. Bound phage particles were detected using an HRP-conjugated anti-M13 monoclonal antibody. The reaction was developed with ABTS as the substrate and measured with a Bio-Rad ELISA reader (OD490).

### DNA sequencing

Single strand phage DNA was prepared with 20% polyethylene glycol-8000 (PEG)/NaCl according to the random phage display 12-mer peptide library kit. Prepared DNA amounts were verified by EtBr2/0.8% agarose gel under UV illumination. DNA sequencing was done by Sangon (ShangHai, China).

### Peptide synthesizing

The selected peptides (DHTLYTPYHTHP (designated as 12P) and corresponding scrambled control peptide PHYTPTYTDHHL (designated as control)) were synthesized (HuaChen, China). Peptide concentrations were calculated based on OD280. The chemically synthesized 12P was conjugated to keyhole limpet hemocyanin (KLH) by GA to form the KLH-12 peptide (KLH-12P). Conjugation procedures were as follows: the peptide was mixed with the carrier protein solution in PBS (1 mg/ml, pH 7.4) at a peptide to KLH ratio of 40:1. The same volume of GA solution was added drop-by-drop to the stirring peptide/protein mixture, which was then incubated at 4°C for 1 h. Uncoupled peptides were removed by size exclusion chromatography, using Sephadex G-25 (Pharmacia, USA).

### Dot blot assay

Avastin was used in a dot blot assay to measure conjugate binding capacity. The conjugate vaccine protein (1 μg) was dotted onto a nitrocellulose membrane. Blot strips were then incubated with Avastin (1 μg) or control Ab. Bound Abs were detected with an alkaline phosphatase-conjugated secondary antibody and western blue as the substrate (Promega, USA).

### VEGF competing assay

The 96-well plate was coated with aliquots (100 μl) of Avastin at 4°C overnight. After blocking with 1% BSA at 37°C for 2 h, the plates were washed three times with PBST. The plate was then incubated with 0, 100, 200, 400 or 800 ng of VEGF and 800 ng biotin-labeled 12P per well at room temperature for 1 h. The plates were washed again and HRP-conjugated streptavidin was then used to act with OPD.

### Immunization of BALB/c mice

Four groups (n = 6 per group) of BALB/c mice were immunized by repeated subcutaneous injections of50 μg of DHTLYTPYHTHP-KLH, control peptide-KLH, KLH alone, or physiological saline on days 1, 15 and 29. Mice were boosted (i.p.) on day 43 and sacrificed on day 53 for serum analysis. The complete Freund adjuvant/incomplete Freund adjuvant (CFA/IFA) (SIGMA, USA) was used as an adjuvant in all groups.

### Antiserum titer determination

Titers of sera from the immunized BALB/c mice against VEGF (Sigma, USA) were determined via ELISA. The 96-well plates were incubated with 50 ng VEGF/per well in 0.1 M NaHCO_3_ overnight at 4°C, pH 8.6. Wells were blocked with 1% BSA in PBS (pH 7.4, 2 h, 4°C). After washing the bound antigen with PBS–0.3% Tween 20, antiserum was added to the wells and incubated for 1 h at 37°C, followed by washing and incubation with the second antibody. The color was developed using ABTS (Sigma, USA) as the substrate. The absorbance was measured at 490 nm.

### Antibody purification

Total serum was collected at day 53 for purification. Ammonium sulphate was added to serum (50% saturated). Precipitates were separated by centrifugation, dissolved in PBS buffer and dialyzed overnight to remove ammonium sulphate. Then DEAE chromatography (GE, Sweden) was applied and eluted fractions related to diagram’s peak were collected and after precipitation with ammonium sulphate were used for G-50 Sephadex gel filtration chromatography (GE, Sweden). Elutions were collected at a stream velocity of 10 ml per hr and optical absorption was read at 280 nm.

### Functional assays of antiserum

Matrigel (Collaborative Biomedical, Bedford, MA) was added (250 μL) to each well of a 24-well plate and was left to polymerize at 37°C for 1 h. HUVECs were seeded onto the plate at a density of 50,000 cells per well and treated with the antiserum dilution at 37°C for 8 h. For the proliferation assay, HUVECs were seeded onto the plate and cultured as monolayers at 37°C in conditioned RPMI 1640 media supplemented with 5 ng/ml of VEGF. After reaching 80% confluence, antibodies purified from the antisera were added and the plates were incubated for 72 h. Then MTT assay was used to assess cell activity. For the migration assay, HUVECs were seeded onto the upper wells of a Millicell Insert in conditioned medium containing antibodies purified from antisera and incubated for 24 h at 37°C. Avastin was used as the positive control. Cell migration was measured by counting the number of migrated cells in five random non-overlapped fields at 100× magnification.

### Statistical analysis

Data were analyzed by Student’s t test. Results were expressed as the means ± standard deviations (SD). A p (probability) value < 0.05 was considered to be significant.

## Competing interests

The authors declare that they have no competing interests.

## Authors’ contributions

YR performed the screening of the phage library. ML and XQ contributed the majority of the work except for the screening of the phage library. KZ purified the antisera. XX participated in the ELISA and DotBlot assays. CZ and QH performed animal experiments. WZ and YZ participated in the design of the study and performed the statistical analysis. WL participated in the coordination and wrote the manuscript. All authors read and approved the final manuscript.

## Supplementary Material

Additional file 1: Figure S1 3D images of the results from MimoPro. The patch with the highest score is selected as a potential candidate for the native epitope. (A) In section "Candidate Epitope", all potential amino acids are listed and each amino acid is printed in the format of [Single letter identifier] [No. in chain]. In the section "Alignment for each mimotope", the resultant alignments for individual peptide sequences are tabulated. 3D analysis (B) 12P, (C) NHFGKFLDALAG and (D) M074_D12 are shown. The candidate epitopes of 1VGH are shown in the shape of spacefill and cpk color format with the rest amino acids in backbone. Click here for file
