# Supplementary material for: Mimotope vaccination for epitope-specific induction of anti-VEGF antibodies
Source: BMC Biotechnol. 2013 Sep 27;13:77. doi: 10.1186/1472-6750-13-77 (PMC3849980; doi:10.1186/1472-6750-13-77)
Supplement: Additional file 1: Figure S1 — 3D images of the results from MimoPro. The patch with the highest score is selected as a potential candidate for the native epitope. (A) In section "Candidate Epitope", all potential amino acids are listed and each amino acid is printed in the format of [Single letter identifier] [No. in chain]. In the section "Alignment for each mimotope", the resultant alignments for individual peptide sequences are tabulated. 3D analysis (B) 12P, (C) NHFGKFLDALAG and (D) M074_D12 are shown. The candidate epitopes of 1VGH are shown in the shape of spacefill and cpk color format with the rest amino acids in backbone. [file 1472-6750-13-77-S1.pdf]

A

Candidate Epitope:

A1 R2 R2 K26 A1 R2 Q3 Q3 R2 E4 K26 P6 A1 R2 Q3 V19 A1 V19 A1 Q3 A1 R2 E4 Q23 R2 Q3 Q23 A1 R2 E4 V19 R2 Q23 A1 R2 Q3 Q3 D21 P22 T24

Alignments for each mimotope:

Resulting Path	Mimotope	score
D21 Q23 T24 Q23 Q3 Q3 Q3 A1 R2 Q3 E4 P6	DHTLYTPYHTHP	23
D21 Q23 T24 A1 Q23 Q3 Q3 Q3 A1 R2 A1 Q3	NHFGKFLDALAG	17
--- --- E4 Q3 R2 E4 P6 A1 E4 ---	WLEMHWPAHS	14

B

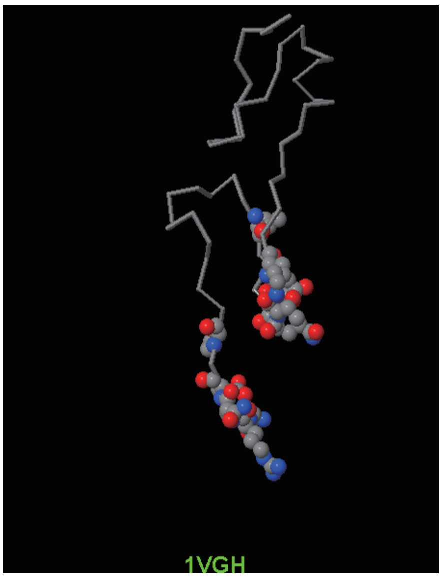

C

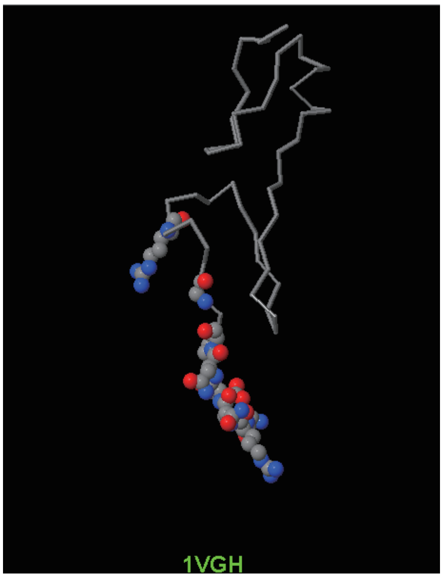

D

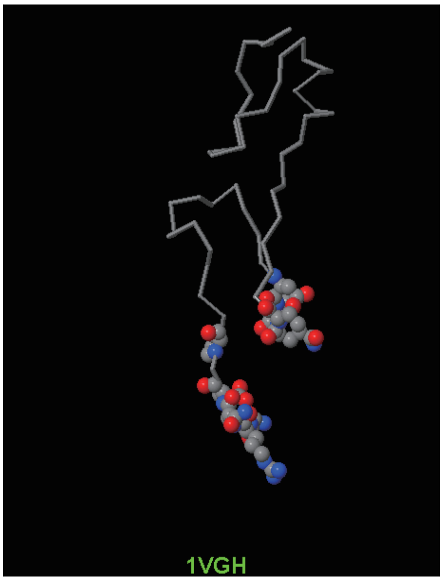

Figure S1. 3D images of the results from MimoPro. The patch with the highest score i selected as a potential candidate for the native epitope. (A) In section “Candidate Epitope”, all potential amino acids are listed and each amino acid is printed in the format of [Single letter identifier][No. in chain]. In the section “Alignment for each mimotope”, the resultant alignments for individual peptide sequences are tabulated. 3D analysis (B) 12P, (C) NHFGKFLDALAG and (D) M074_D12 are shown. The candidate epitopes of 1VGH are shown in the shape of spacefill and cpk color format with the rest amino acids in backbone.
